# The opiate dosage adequacy scale for identification of the right methadone dose—a prospective cohort study

**DOI:** 10.1186/s40360-016-0058-9

**Published:** 2016-04-07

**Authors:** Stephan Walcher, John Koc, Volker Reichel, Frank Schlote, Uwe Verthein, Jens Reimer

**Affiliations:** Concept, Addiction Medicine, Kaiserstr. 1, D-80801 Munich, Germany; Psychiatry and Addiction Medicine, Stockholmer Str. 51, D-28719 Bremen, Germany; General Medicine, Kaiserplatz 17, D-53113 Bonn, Germany; Turmstrasse, Turmstrasse 76, D-10551 Berlin, Germany; Centre for Interdisciplinary Addiction Medicine, Hamburg University, University Medical Center Hamburg-Eppendorf, Martinistrasse 52, D-20246 Hamburg, Germany; Health North Bremen, Kurfürstenallee 130, D-28211 Bremen, Germany

**Keywords:** Opioid dependence, Substitution treatment, Dosing, Outcomes

## Abstract

**Background:**

Opioid maintenance treatment with methadone is regarded as gold standard in the therapy of opioid dependence. Identification of the ‘right’ methadone dose, however, remains challenging. We wanted to explore if the Opiate Dosage Adequacy Scale (ODAS) is a helpful instrument in methadone titration.

**Methods:**

Within this 12-months prospective naturalistic cohort study patients in stable maintenance treatment with methadone (Eptadone®) were included. Sociodemographic and clinical data were gathered at baseline, and months 3, 6, and 12. At the same points in time, the instruments ODAS, European Addiction Severity Index (EuropASI), and Derogatis Interview for Sexual Functioning-Self Report (DISF-SR) were applied.

**Results:**

Five hundred fifteen patients were enrolled, 129 patients prematurely terminated substitution treatment (treatment failure), in 108 patients substitution medication was changed, likely due to bitter taste of Eptadone®. Complete longitudinal ODAS and EuropASI data sets were available for 229 patients. The frequency of adequate methadone doses (ODAS) increased (60.9 % at baseline, 85.3 % at month 12) as well as the average daily methadone dose (63.8 (±30.8) mg/day at baseline to 69.6 (±36.0) mg/day at month 12). Inadequacy of methadone dose was not associated with treatment failure (RR 1.019; CI 95 % 0.756–1.374). Addiction severity decreased statistically significantly. Compared to adequately dosed patients, inadequately dosed patients benefited more, in that they showed greater improvements in ODAS scores, had higher increases in methadone dose, and partially experienced more advanced sexual functioning.

**Conclusion:**

Application of ODAS was associated with improved methadone dose adequacy and addiction severity parameters as well as increased methadone doses. Its usefulness should be corroborated in a controlled trial.

## Background

Dependence on illegal opioids is a severe chronic illness, which is associated with relevant increased morbidity, functional impairments, social disintegration and premature mortality [1–3]. Opioid maintenance treatment (OMT) comprises the replacement of an illegal opioid by a prescribed opioid, favorably with good μ-receptor activity and longer half-life to avoid euphoric and withdrawal symptoms. OMT reduces mortality, illicit drug use, frequency of injecting, HIV-transmission, and criminal activity whilst improving social integration [1, 3–6]. Methadone and buprenorphine represent the medications most commonly used in OMT, and both have widely proven effectiveness. Methadone seems to have some advantages in terms of treatment retention when doses are in the range of 60–100 mg per day [7, 8]. Aside from the absolute methadone dose, also the perception of dose inadequacy can lead to treatment non-adherence [9]. Consequently, an absolute methadone dose cannot serve as a sole indicator of sufficiency and should be related to clinical and patient reported parameters. In this context, Trujols and colleagues suggested distinguishing between the concepts *holding dose*, *dose adequacy*, *satisfaction with methadone as a medication*, and *satisfaction with treatment* [10]*.* The latter two dimensions represent subjective phenomena, while the first two constructs include subjective and objective phenomena, with *dose adequacy* as a construct that can be measured by a validated instrument, the Opiate Dose Adequacy Scale (ODAS) [11, 12]. An *adequate dose* is defined as the amount of methadone, which allows the patient (a) to abstain from heroin use or only use heroin occasionally, (b) not to experience continuous opioid withdrawal or only mild withdrawal symptoms, (c) not to experience frequent episodes of heroin craving or only mild episodes, (d) in case of heroin use, to experience no or little effect of heroin (cross-tolerance), and (e) not to show signs and symptoms of overmedication or only to a very small extent [10]. Compared to *adequate doses*, sub-therapeutic doses (<40 mg methadone per day) are associated with more severe symptoms in aforementioned dimensions.

Scientific literature and scientifically based medical guidelines (i.e. World Health Organization) do not recommend to initiate OMT with a primary abstinence goal (maintenance to abstinence), however, the German Physician Chamber and treatment regulations (Narcotics Act, *Betäubungsmittelverschreibungsverordnung, BtMVV*) still regard abstinence as a core target [13–15]. Consequently, a relevant number of German physicians deliver OMT with high abstinence orientation and lower substitution doses, despite the fact that stable abstinence is a rare outcome [16]. In a large German longitudinal clinical-epidemiological study over 6 years in around 2700 substituted patients average daily substitution doses were around 75 mg for d,l-methadone, 55 mg for l-methadone, and 7 mg for buprenorphine, around 40 % of patients received methadone doses < 60 mg per day [17].

To examine dose adequacy in OMT in Germany, the present study was conducted. Baseline data are reported elsewhere and point to high frequencies of inadequate dosing (40 % of the sample) according to ODAS [18]. On average, patients with inadequate doses received higher doses (70.6 (±33.0) mg methadone per day) compared to adequately dosed patients (57.8 (±27.5) mg methadone per day) and suffered from a more pronounced addiction severity according to the European Addiction Severity Index (EuropASI) [18]. In this context, an instrument like ODAS could serve as a reliable tool to guide healthcare providers and patients to identify a methadone dose, which meets the individual’s needs. Analyses of longitudinal data aimed to explore whether inadequate dosing leads to treatment drop-out, how dose adequacy is associated with treatment outcome in general, and whether frequencies of inadequate dosing, addiction severity or sexual functioning changed over time. We hypothesized, that inadequate dosing leads to increased treatment drop-out, that addiction severity improves over time, and that sexual dysfunction is more pronounced in adequately dosed patients. Finally we wanted to explore, whether the use of ODAS could be associated with increased frequencies of adequate dosing.

## Methods

Within an observational, prospective cohort study (Prospective observational study to assess the efficacy and tolerability of Eptadone® in heroin addicted patients undergoing a methadone maintenance treatment, METHO.DE study), 14 experienced substitution centres in Germany consecutively recruited patients with opioid dependence (F11.2) according to the International Classification of Diseases-10 (ICD-10) (International Classification of Mental Disorders, 2010). Further inclusion criteria comprised age 18 or older, maintenance treatment with methadone (Eptadone®) at least 1 month prior to study entry and signed informed consent Methadone was administered once daily orally. Patients were excluded from study entry in case of inability to follow the study plan, decompensated mental disorders, non drug-related epileptic seizures and acute or severe somatic diseases. Over the one-year study period, visits were scheduled at baseline (t_0_), 3 months (t_1_), 6 months (t_2_), and 12 months (t_3_). The baseline visit included a complete medical anamnesis and physical examination, as well as recording of routine laboratory testing if it was performed within 2 months prior the baseline visit. Additionally, all visits comprised the European Addiction Severity Index (EuropASI), the Opiate Dosage Adequacy Scale (ODAS), and the Derogatis Interview for Sexual Functioning-Self Report (DISF-SR). These diagnostic instruments were handed out by a nurse specialist. Patients filled out the instruments in a secluded room during waiting time.

The EuropASI is a validated and reliable instrument that covers drug use-related consequences in social, health and legal dimensions (i.e. somatic health, work and economic situation, alcohol use, legal situation, family and relationships, and mental health) [19, 20]. It was applied by trained interviewers, and reported scores reflect the interviewer severity ratings. The ODAS is a validated instrument to measure dose adequacy of methadone medication in maintenance treatment, which has been adapted to the German background [11, 12]. ODAS consists of six dimensions and comprises the areas heroin consumption, narcotic blockade, opiate withdrawal syndrome mental and somatic, craving for heroin, and methadone overdosing. Items have to be filled out either by the physician based on the patient anamnesis or by the patient himself. Scores range between 1 (worst) and 5 (best). In this study, patients were regarded as not adequately dosed, if they reached a score of 3 or less in any ODAS dimension at *any* visit. On the other hand, patients were considered adequately dosed, if they reached a score of 4 or higher in every item in *every* visit.

For comparative analyses two groups of patients were formed. Doses were regarded as adequate, if scores of 4 or 5 were yielded in each item in *every* visit. Patients were assigned to the inadequate dose group, if their ODAS score failed to meet adequate dosing threshold at least once throughout the study. The DISF-SR represents a validated and reliable 25-item self-report instrument, which measures sexual functioning in five dimensions (i.e. sexual cognition/fantasy, sexual arousal, sexual behavior/experience, orgasm, and sexual drive/relationship) [21].

The study was approved by the ethics committee of the Physician Chamber Hamburg (Reference PV3468), Germany, and by all responsible physician chamber ethics committees outside the state of Hamburg. The study was conducted in accordance with the declaration of Helsinki. Patients provided informed written consent and could withdraw from the study at any time without providing reasons and without negative consequences for further treatment. Patients were included in the study between September 2010 and February 2012.

Data analysis was performed with the SPSS 20 statistical package. Data is presented in a descriptive way, statistical analyses were performed to stratify by dose adequacy and in case of sexual functioning by gender. The Mann—Whitney test, student *t*-test, or Chi^2^-test were used for analysis of statistical significance. To determine the association of dose adequacy, age, and gender, relative risks were computed. The level of significance was set at *p* < .05.

## Results

**Fig. 1:**
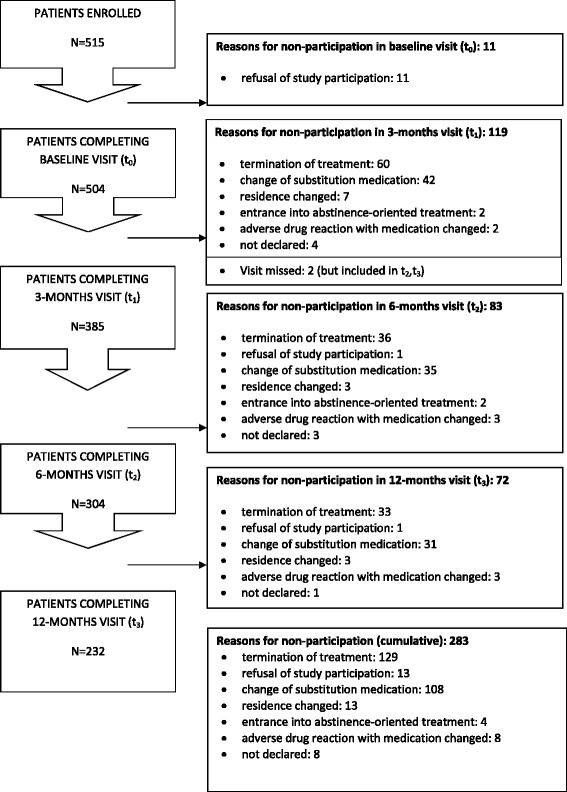
Study flow

Five hundred fifteen patients were enrolled in the study. Eleven patients withdrew their consent before the baseline visit. Following relative data are calculated with *n* = 504 patients participating in the baseline visit as parent population. Two hundred thirty-two patients (46.0 %) regularly completed the study with the 12 months (t_3_) visit, 129 patients (25.6 %) prematurely terminated substitution treatment (treatment failure), in 108 patients (21.4 %) the substitution medication was changed, likely due to dislike or bitter taste of Eptadone®, and in eight cases the substitution medication was changed due to adverse drug reactions. The remaining drop-outs were due to changed residence, entrance into drug free treatment, or unknown (Fig. 1). In three patients, data for ODAS and EuropASI were incomplete, consequently longitudinal data on clinical parameters and ODAS as well as EuropASI were analyzed in 229 patients. However, complete data including DISF-SR were available for 160 patients only. At baseline, patients were on average 36.5 (±9.2) years old, three out of four participants were male, and they were predominantly Caucasian, with high frequencies being single and living alone, with low formal education and high unemployment rates (Table 1).

**Table 1:** Baseline sociodemographic characteristics

Average age, years (SD) (*n* = 515)	36.5 (9.2)
Male gender, % (*n* = 515)	74.4
Ethnicity, % (*n* = 504)	
Caucasian	97.2
African	0.6
Asian	2.2
Marital status, % (*n* = 505)	
Single	75.6
Married	12.1
Divorced	12.3
Living situation, % (*n* = 505)	
Alone	68.5
With family	25.1
Homeless	0.8
Other	5.5
Education, % (*n* = 505)	
8–9 years school	63.2
10 years school	23.6
13 years school	5.1
University degree	1.8
Other	6.3
Vocational situation, % (*n* = 505)	
Unemployed	59.0
Employed	34.3
Other (student, homemaker)	6.7

**Fig. 2:**
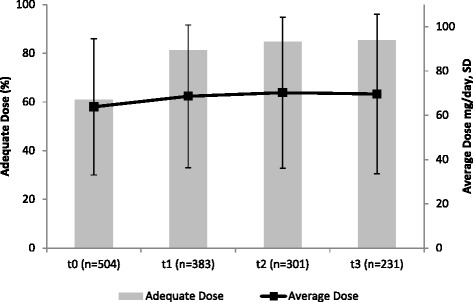
Frequency of adequate methadone dose (ODAS) and average methadone dose by time (t_0_: baseline, t_1_: 3 months, t_2_: 6 months, t_3_: 12 months)

**Fig. 3:**
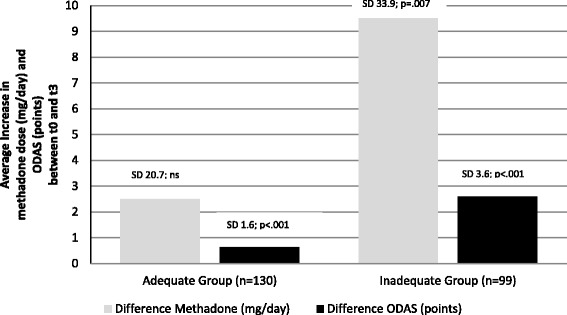
Changes in methadone dose and ODAS total score between baseline (t_0_) and 12 months (t_3_) by adequacy group

According to ODAS, 60.9 % of a total of 504 patients received adequate methadone doses at baseline, this frequency increased to 81.2 % at t_1_ (total *n* = 383), to 84.7 % at t_2_ (total *n* = 301), and 85.3 % at t_3_ (total *n* = 231). In a similar direction, the average methadone dose for the whole group increased from 63.8 (±30.8) mg/day at baseline, to 68.6 (±32.2) mg/day at t_1_, and 70.2 (±34.1) mg/day at t_2_, with a slight decrease at t_3_ with 69.6 (±36.0) mg/day (Fig. 2). Over time (t_0_ to t_3_), adequacy of methadone dosage increased statistically significant in all ODAS domains, except methadone overdosing. Increase was reflected distinctly in the ODAS global score (+1.5 (±2.8), *p* < .001), and to a smaller extent in the areas heroin consumption (+.1 (±.6), *p* < .001), narcotic blockade (+.2 (±.7), *p* < .001), physical opiate withdrawal syndrome (+.4 (±.1.1), *p* < .001), mental opiate withdrawal syndrome (+.4 (±1.0), *p* < .001), and craving for heroin (+.4 (±.9), *p* < .001). Increase of ODAS scores was statistically significant in both the adequate (global score + .64 (±1.6), <.001) and the inadequate group (+2.6 (±3.6), *p* < .001), however, it was more pronounced in the inadequate group. Daily methadone dose increased both in the adequate and inadequate group over time, however increase between t_0_ and t_3_ was statistically significant in inadequately dosed patients only (+9.5 (±33.9) mg methadone per day, *p* = .007; adequate group +2.5 (±20.7) mg methadone per day, *p* = .171) (Fig. 3). The average methadone dose in the adequate group at t_3_ was 66.5 (±35.8) mg methadone per day, in the inadequate group this number was 87.8 (±31.8) mg methadone per day, frequencies of patients receiving less than 40 mg methadone per day at t_3_ were 28.4 % for the adequate (t_0_ 30.3 %) and 11.8 % for the inadequate (t_0_ 25.4 %) group (statistically significant at t_3_*χ*^2^ 6.485, df = 1, *p* < .05).

Inadequacy of methadone dosage according to ODAS was not associated with treatment failure; the relative risk for patients who were at least once inadequately dosed to fail in substitution treatment was 1.019 (CI 95 % 0.756–1.374) compared to those, who did not fail. Likewise, the log rank test for retention in the study by dose adequacy did not show a statistically significant difference (*χ*^2^ .141, df = 1; *p* = .707). Additional tests explored the role of age and gender with regards to treatment failure. In this context, being younger than 40 years was associated with a higher risk for treatment failure (RR 1.68; CI 95 % 1.173–2.410; log rank test *χ*^2^ 8.512, df = 1; *p* = .004), whilst gender was not associated with treatment failure (RR 0.97; CI 95 % 0.693–1.360; log rank test *χ*^2^ 0.060, df = 1; *p* = .807).

**Fig. 4:**
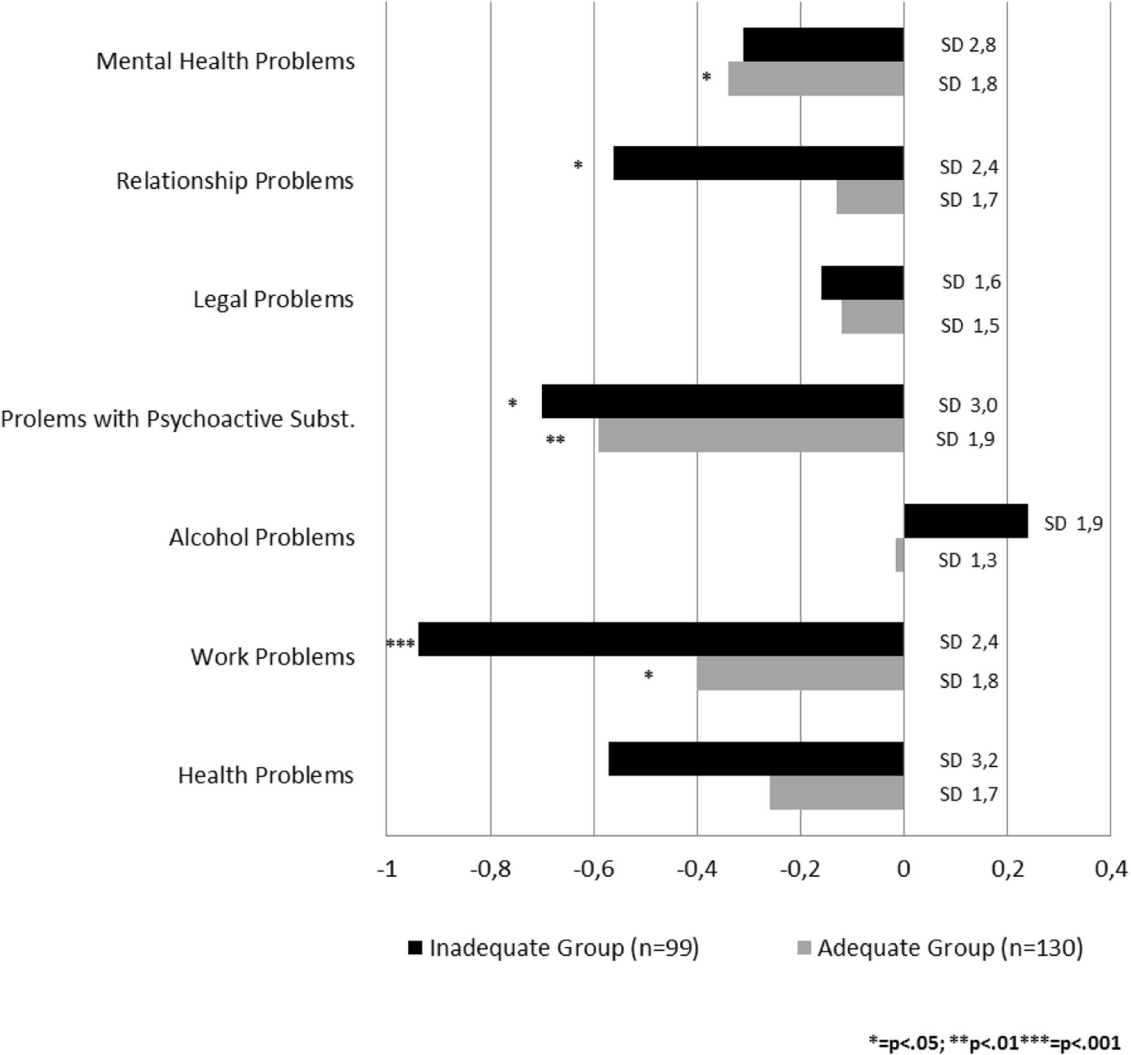
Changes between baseline (t_0_) and 12 months (t_3_) in EuropASI score

Addiction severity according to EuropASI decreased during study participation, but differed by group. Significant reductions in the adequate group were found for mental health problems (−0.34; ±1.8; *p* = .033), problems with psychoactive substances (−.59; ±1.9; *p* = .001), and work problems (−.39; ±1.8; *p* = .012), whereas the areas relationship problems (−.56; ±2.4; *p* = .023), problems with psychoactive substances (−.72; 3.0; *p* = .018), and work problems (−.94; ±2.4; *p* < .001) improved significantly in the inadequate group (Fig. 4). Disparities in addiction severity between the adequate and non-adequate group with statistically stronger problem load in all EuropASI domains in the inadequate group at baseline (t_0_), resolved during study participation.

Sexual functioning improved in men and women over time (t_0_–t_3_), but failed to reach statistical significance, except for sexual cognition/fantasies (−2.5 (±11.9), *p* = .026) and sexual drive in men (−1.6 (±5.9), *p* = .005). The subgroup analysis revealed no statistically significant improvements in adequately dosed patients. However the domain sexual drive improved in inadequately dosed men (−2.0 (±6.1), *p* = .032) and sexual behavior/experience improved in inadequately dosed women (−3.6 (±5.2), *p* = .007).

## Discussion

This prospective cohort study examined retention, dose-adequacy, addiction severity, and sexual functioning within a 12-months opioid maintenance treatment in more than 500 patients in experienced German substitution centres. OMT failed in 25.6 % of patients, another relatively high proportion of patients (21.4 %) dropped out from the study due to change of the substitution medication, but remained in OMT. In this context, it is noteworthy, that the reported bitter taste of Eptadone® contributed substantially to the demand for a medication switch. Formulation of drugs, especially for long-term treatment, influences adherence and should be considered in OMT [22].

Overall, the treatment retention rate in our study is comparable to retention rates of OMT in Germany, which are around 75 % after 1 year. German retentions rates are at the upper end of the international span, which ranges between 22.7 % at 6 months in Iran and 79.6 % at 13 months in Israel [23–25]. High retention rates in Germany may be regarded as an indicator for good quality delivery of OMT. Contrary, a surprisingly high number of patients was inadequately dosed upon study entry. Abstinence orientation in German opioid substitution regulations may contribute to restrained dosing [16, 26, 27]. However, inadequate methadone doses according to ODAS at baseline did not go along with premature treatment drop-out. This finding is not in line with corroborated study data [7, 8]. We assume that assessment with ODAS led to a better consideration of patients’ needs which is reflected in increased methadone doses and increased frequencies of adequate dosing. Patients may have remained in OMT, because they were aware that their concerns were taken into account. On the health care providers’ side, application of ODAS may have paved way for a dose increase, as this need was documented by a standardized inventory.

Treatment effectiveness is also reflected by improvements in addiction severity, which was more pronounced in the group of inadequately dosed patients. Due to a more sufficient methadone dose, we expected stronger addiction severity improvements in adequately dosed patients. However, more pronounced addiction severity in inadequately dosed patients at baseline together with more intense methadone dose adaptions may have contributed to stronger improvements of addiction severity in inadequately dosed patients [18]. This finding is relevant for clinical practice, as it shows, that inadequately dosed patients may derive relevant benefit from dose adaptations. Increased adherence associated with adequate dosing might act as intermediary to improved functional outcomes [9]. It is noteworthy that, in inadequately dosed patients, work problems improved the most, followed by problems with psychoactive substances. This finding underlines the importance of OMT in social rehabilitation [3]. On the other hand, increased alcohol problems in inadequately dosed patients are of concern and call for an integrative addiction treatment approach, which takes the variety of substances consumed into account.

Higher methadone doses have been associated with sexual dysfunction [28]. We did not find a positive association between methadone dose and sexual dysfunction. On the contrary, sexual function improved statistically (although not significantly) during study participation with increasing methadone doses. Considering the improvements of the drug use problem and overall life situation (EuropASI), we assume that general health and psychosocial aspects play an important role in sexual functioning. When discussing sexual functioning with the patient, aforementioned issues should be considered besides methadone dose [18, 29, 30].

Finally we wanted to explore whether ODAS is a useful tool for identifying the ‘right’ methadone dose. From a methodological viewpoint, an observational study without control group does not deliver hard facts, but indices, which may or may not lead to the proposal of a controlled clinical study. When summarizing the findings, that a) ODAS scores improved, b) daily doses of methadone increased, whilst c) frequencies of sub-therapeutic doses (<40 mg/day) decreased, and d) treatment proved to be successful according to core outcome parameters (EuropASI), one can conclude, that opioid maintenance treatment is effective and adequate dosing can further improve these effects. On the background of the German (legal) treatment framework with its inherent abstinence orientation, ODAS may serve as an instrument, which provides standardized clinical parameters to guide dose titration. In the future, this hypothesis should be examined in a controlled clinical trial.

## Conclusion

Identification of an adequate methadone dose in opioid substitution treatment is a frequent and difficult challenge in daily practice. Quarterly application of the ODAS was associated with a clinically relevant increase in adequately dosed patients. At the same time, addiction severity decreased and sexual functioning improved partially. Low-frequency use of ODAS in substitution treatment may serve as useful approach to support adequate methadone doses in substitution treatment.

### Availability of supporting data

Supporting data are available in form of an extended study report to the ethics committee of the Physician Chamber Hamburg, Germany. This report is available upon request, which should be addressed to the corresponding author.
